# Survival, functional outcome and satisfaction of first revision total knee arthroplasty at a mean eleven-year follow-up

**DOI:** 10.1007/s00590-022-03206-1

**Published:** 2022-01-18

**Authors:** Andreas Hecker, Hans-Jürg A. Pütz, Sebastian Wangler, Frank M. Klenke

**Affiliations:** Department of Orthopaedic Surgery and Traumatology, Inselspital, Bern University Hospital, University of Bern, Freiburgstrasse 4, 3010 Bern, Switzerland

**Keywords:** Knee, Arthroplasty, Revision, Cause, Outcome, Satisfaction

## Abstract

**Purpose:**

Providing long-term outcome data after rTKA and compare one- versus two-stage and septic versus aseptic revisions.

**Methods:**

This study represents a single-center retrospective study of first rTKAs performed for any reason with a final follow-up of a minimum of five years. Outcome parameters included stability assessment ROM, radiologic assessment, HSS score, KSS score, OKS score, EQ-5D-3L and VAS. 44 patients were included in the study. Subgroups analysis of one- versus two-stage revision and septic versus aseptic revision was performed.

**Results:**

The leading causes of rTKA in this mean 11 year follow-up study were aseptic loosening (36%) and periprosthetic joint infection (27%). At the final follow-up, there was a 89% survivorship of the implants. Patients showed a ROM of 114 ± 13°, HSS score of 78 ± 12, KKS objective score of 77 ± 16, KSS expectation and satisfaction score of 32 ± 11, KSS functional activity score of 50 ± 20, OKS of 30 ± 9, VAS of 53 ± 25 and EQ-5D index of 0.649. Functional outcome scores were not significantly altered in the analyzed subgroups.

**Conclusions:**

In our 11 years follow-up, we obtained 89% implant survivorship. Measurements regarding functional outcome and pain showed results in the medium range of the respective scores, while patient satisfaction lay in the upper third. No significant differences in outcome scores between one- and two-stage revisions and septic versus aseptic revisions were observed.

*Level of Evidence* Level III, retrospective cohort study.

## Introduction

Total knee arthroplasty (TKA) represents a well-established approach for the treatment of advanced degenerative arthritis. Based on register data available in many countries, the reported survivorship of primary TKA is at 82% at 20-year and 70% at 25-year follow-up [8]. Fostered by these good long-term results, indications for TKA have been expended in the past years. Especially the age at primary implantation has dropped, resulting in a more active patient population requesting a higher performance of the used implant. The number of TKA procedures is therefore expected to increase by 140% in 2050 [11]. Importantly, patients younger than 65 years will represent the major recipients of primary TKA [18]. The aim of TKA is the long-term relief of pain and restoration of function. Unfortunately, knee replacements fail for various reasons, including aseptic loosening and infection, followed by instability, wear, and pain [6]. In this respect, younger age has been associated with a higher risk of aseptic mechanical failure after TKA. Considering the growing number of performed procedures and expended target population, orthopedic surgeons will be confronted with an increasing number of patients in need of revision TKA (rTKA). Some authors estimate that the number of rTKA will increase by up to 600% in 2030 [18].

While functionality and patient satisfaction following primary TKAs has been investigated thoroughly, data about the outcome after rTKA is limited. Primary TKA has been associated with a satisfaction rate of about 80%. Some studies report similar outcomes following rTKA, while others report limited success [13, 27, 30]. An explanation for these heterogeneous results might be a different overall health status of the respective cohorts. Interestingly, the reason for revision surgery seems to influence the outcome. Revision TKA following aseptic loosening was associated with a better outcome than revision due to instability, malposition, or septic loosening [32].

Despite the indication, revision surgery is always challenging because many factors, including bone loss, ligamentous instability, and soft tissue problems, need to be considered. Late infection represents a challenging situation as it often requires a two-staged revision with primary infect control and secondary rTKA. The patients’ immobilization between the two steps results in loss of muscle and is associated with a prolonged rehabilitation [24]. In general results after rTKA are thought to be worse than after primary TKA due to scaring, a tendency to develop patella baja, muscular imbalance and change of the biomechanics due to revision implants and/or bone loss.

This study aimed to analyze the outcome and implant survival of patients undergoing the first TKA revision, focusing on subjective satisfaction after a minimum follow-up of five years. We hypothesized that functional outcome following one- and two-stage revisions as well as after septic or aseptic revision does not differ after a minimum follow-up of five years.

## Material and methods

This study represents a single-center retrospective study of rTKA performed at our institution between January 2000 and December 2012. The local ethics committee approved the study.

**Fig. 1:**
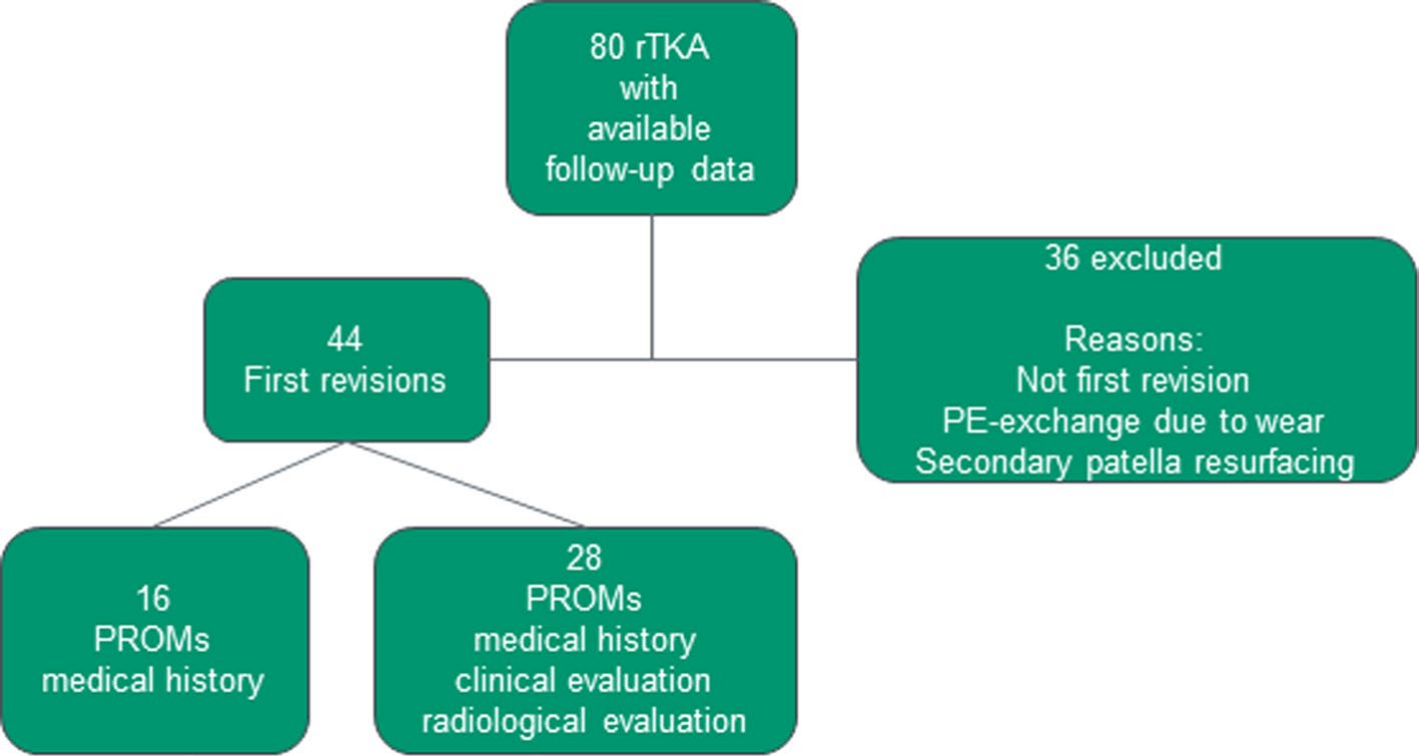
Patient selection chart

Inclusion criteria were first rTKA performed for any reason with a well-documented final follow-up of a minimum of five years and available outcome scores (Hospital of Special Surgery Score (HSS), Knee Society Score (KSS), Oxford Knee Score (OKS), EQ-5D-3L, Visual Analog Scale (VAS)) [5, 7, 12]. Exclusion criteria were re-revisions (if first revision was not performed at our institution during the observational period), only exchange of the polyethylene inlay due to wear and secondary patellar resurfacing. Database evaluation revealed a total of 80 rTKAs eligible for this study. Thirty-six patients had to be excluded after application of the above mentioned exclusion criteria. Finally, 44 patients matched the inclusion criteria and were included in the study (Fig. 1).

The medical history, including the surgical report, was analyzed. The reason for revision, time to revision and the implant model used for revision were documented. Age, American Society of Anesthesiologists (ASA) score, height, and weight were raised. Clinical evaluation was performed focusing on stability and range-of-motion (ROM). Radiographic evaluation, including long-leg, standing anteroposterior and lateral views, was performed to detect implant loosening signs and evaluate the leg axis. Loosening was defined as a gap larger than 2 mm in the bone-cement- or bone-implant-interface [17]. In the OKS, the best value is 12 and the worst 60. All other scores range from 0 to their maximum, with the latter representing the best reachable score.

A subgroup of the cohort (*N* = 16) was not available for physical and radiological examination at the final follow-up, but outcome scores could be raised and surgical history was present; therefore, those patients were also included for the respective analysis.

### Statistical analysis

Statistical analyses were conducted in SPSS (IBM SPSS Statistics, Version 25 for Windows) with *P<* 0.05 considered statistically different. Data normality was checked with the Kolmogorov–Smirnov test. When the normality assumption was satisfied, a student *t*-test was chosen to compare different subgroups. Data are given as mean and standard deviation. Kaplan–Meier survival was calculated with endpoints re-revision and implant survival.

## Results

**Fig. 2:**
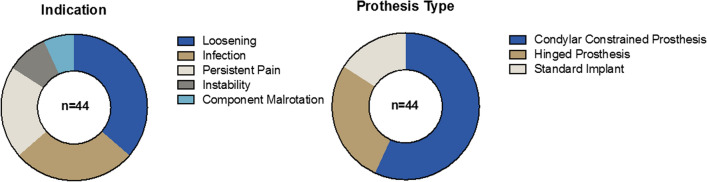
Indications for revision TKA and implant type at the final follow-up. In the left pie-chart, the indications that lead to the first revision TKA are displayed. The biggest group was loosening (blue) followed by infection (brown). In the right pie-chart the implant type at the final follow-up is shown. The biggest group consists of condylar constrained implants (blue) followed by hinged implants (brown) (color figure online)

The present retrospective single-center study reports the outcome of 44 patients following first rTKA (50% female, average age 63 ± 8 years at rTKA). The right knee was affected predominantly in 61% of the cases. Regarding the overall population health status, 33 cases were classified ASA 2 and 11 ASA 3. The body mass index (BMI) was 29 ± 5 kg/m^2^. The mean follow-up was 11 ± 3 years. The mean time between first TKA and revision surgery (first revision) was 5 ± 6 years. All implants used for the primary TKA were standard cruciate retaining or posterior stabilized implants. The reasons for revision were loosening (36%), infection (27%), persistent pain (20%), instability (10%), and component malrotation (7%). In 24 cases, a one-stage exchange was performed, while in 20 cases, a two-stage exchange was chosen. In eight of the two-stage exchanges, a preoperatively suspected infection could not be confirmed by intraoperative tissue samples and sonication. In 25 cases a condylar constrained implant was used, in 12 cases a hinge design was chosen and in seven cases a standard implant was utilized (Fig. 2, Table 1).

**Table 1:** Demographic data, radiological and functional outcome of all included patients

N		44
Gender	Female	50% (22/44)
	Male	50% (22/44)
Age at index surgery (years)		58 ± 9 (44)
Age at rTKA (years)		63 ± 8 (44)
Age at follow-up (years)		74 ± 9 (44)
ASA classification	ASA 1	0% (0/44)
	ASA 2	75% (33/44)
	ASA 3	25% (11/44)
	ASA 4	0% (0/44)
BMI (kg/m^2^)		29 ± 5 (44)
Affected side	Right	61% (27/44)
	Left	39% (17/44)
Time to revision after index TKA (years)		5 ± 6 years (44)
Cause of rTKA	Loosening	36% (16/44)
	Infection	27% (12/44)
	Persistent pain	20% (9/44)
	Instability	10% (4/44)
	Component malrotation	7% (3/44)
Approach	One-stage	55% (24/44)
	Two-stage	45% (20/44)
Re-rTKA		14% (6/44)
Re–Re-rTKA		33% (2/6)
Mean follow-up (years)		11 ± 3 (44)
Implant at follow-up	Condylar constrained prosthesis	57% (25/44)
	Hinged prosthesis	27% (12/44)
	Standard implant	16% (7/44)
*Radiological outcome at follow-up*		28
Varus axis (°)		4 ± 0 (2)
Valgus axis (°)		5 ± 3 (26)
Loosening (radiolucent line > 2 mm)		7% (2/28)
*Functional outcome at follow-up*		**28**
ROM (°)		114 ± 13 (28)
Medio-lateral stability		
	No instability	71% (20/28)
	Instability < 5 mm	29% (8/28)
	Instability > 5 mm	0% (0/28)
Antero-posterior stability		
	No Instability	89% (25/28)
	Instability < 5 mm	11% (3/28)
	Instability > 5 mm	0% (0/28)
HSS score		78 ± 12 (28)
	HSS objective	41 ± 5 (28)
	HSS symptoms	39 ± 10 (44)
KSS score		77 ± 16 (28)
	KSS objective	62 ± 15 (28)
	KSS symptoms	16 ± 6 (44)
KSS expectation and satisfaction score		32 ± 11 (44)
	KSS satisfaction	24 ± 9 (44)
	KSS expectation	9 ± 3 (44)
KSS functional activity score		50 ± 20 (44)
	KSS walking and standing	19 ± 9 (44)
	KSS standard activities	17 ± 7 (44)
	KSS advanced activities	7 ± 6 (44)
	KSS discretionary activities	7 ± 5 (44)
OKS (12 = best result, 60 = worst result)		30 ± 9 (44)
EQ-5D VAS		53 ± 25 (44)
EQ-5D Index (Reference: German value set)		0.649 ± 0.173 (44)

ASA = American society of anesthesiologists; BMI = Body mass index; TKA = Total knee arthroplasty; rTKA = Revision total knee arthroplasty; ROM = Range of motion; HSS = Hospital for special surgery score; KSS = Knee society score; OKS = Oxford knee score; EQ-5D = Measure of health-related quality of life

**Fig. 3:**
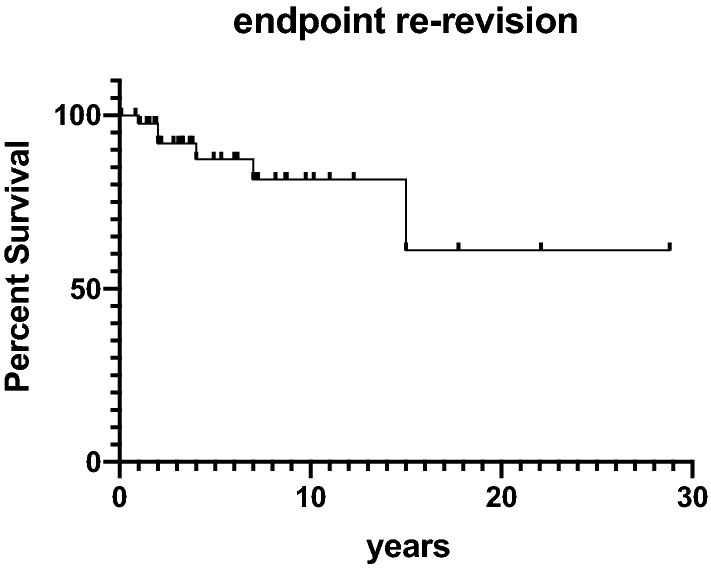
Kaplan–Meier survival with the endpoint re-revision. The *x*-axis gives the follow-up in years and the *y*-axis the percent survival

**Fig. 4:**
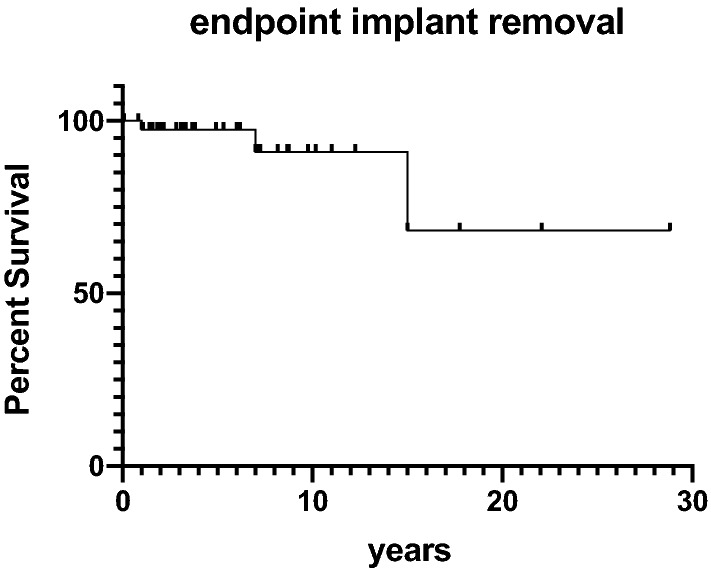
Kaplan–Meier survival with the endpoint implant removal. The *x*-axis gives the follow-up in years and the *y*-axis the percent survival

In six cases (14%), a re-revision was necessary after a mean of 5 ± 5 years. Indications for re-revision with complete implant exchange were infection, trauma and pain without obvious reason in one case each. The implant was retained in three cases of secondary patellar resurfacing. Of the six re-revision cases, two secondary patella resurfacing cases needed a third revision with implant removal due to instability 7 months and loosening 32 months after re-revision. The cumulative Kaplan–Meier implant survival estimations for the endpoint re-revision is displayed in Fig. 3 and for the endpoint implant removal in Fig. 4. The implant survival at a mean of 11 years was 89% in this study.

In 28 cases, complete follow-up with the clinical and radiological examination was available, and in 16 cases, only outcome scores and medical history could be obtained.

### Radiological outcome

Twenty-six patients had a mean anatomical valgus of 5 ± 3°, and two patients had a mean varus axis of 4 ± 0°. In 2 cases, a radiolucent line of more than 2 mm was detected on the radiographs, and therefore loosening was diagnosed.

### Functional outcome

At the final follow-up, 8 patients showed limited medio-lateral instability of less than 5 mm. In 20 patients, no medio-lateral instability was detected. In 3 patients, an antero-posterior instability of less than 5 mm was found, while 25 cases were stable. The mean range of motion was 114 ± 13°.

The mean HSS score was 78 ± 12. The mean HSS symptoms score was 39 ± 10, the mean HSS objective score was 41 ± 5. The mean KSS overall score was 77 ± 16, subdivided in an objective score of 62 ± 15 and a symptoms score (including patients with no objective measurements) of 16 ± 6. The mean KSS expectation and satisfaction score was 32 ± 11. The mean KSS functional activity score was 50 ± 20. The mean OKS score was 30 ± 9. The mean EQ-5D VAS level was 53 ± 25. The mean EQ-5D index was 0.649 ± 0.173.

### Subgroup analysis

Subgroups were analyzed to test for differences regarding one or two-stage revision strategy (Table 2) and septic versus aseptic revisions (Table 3). No significant differences were found regarding functional outcome scores between one- and two-stage approaches. [Table 2; HSS symptoms score (*p* = 0.748), KSS symptoms score (*p* = 0.338), KSS expectation and satisfactions score (*p* = 0.346), KSS functional score (*p* = 0.524), OKS score (*p* = 0.137) and EQ-5D VAS level (*p* = 0.474)].

**Table 2:** Demographic data, radiological and functional outcome one-stage vs. two-stage

		One-stage	Two-stage	*p*
N		24	20	
Gender	Female	50% (12/24)	50% (10/20)	
	Male	50% (12/24)	50% (10/20)	
Age at index surgery (years)		57 ± 10 (24)	58 ± 7 (20)	
Age at rTKA (years)		63 ± 9 (24)	63 ± 8 (20)	
Age at follow-up (years)		74 ± 10 (24)	73 ± 9 (20)	
ASA classification	ASA 1	0%	0%	
	ASA 2	75% (18/24)	75% (15/20)	
	ASA 3	25% (6/24)	25% (5/20)	
	ASA 4	0%	0%	
BMI (kg/m^2^)		29 ± 4 (24)	30 ± 5 (20)	
Affected side	Left	63% (15/24)	60% (12/20)	
	Right	37% (9/24)	40% (8/20)	
Time to revision after index TKA (years)		6 ± 7 (24)	5 ± 4 (20)	
Causes of rTKA	Loosening	29% (7/24)	45% (9/20)	
	Infection	4% (1/24)	55% (11/20)	
	Persistent pain	38% (9/24)	0%	
	Instability	17% (4/24)	0%	
	Component malrotation	12% (3/24)	0%	
Re-rTKA		13% (3/24)	15% (3/20)	
Re–Re-rTKA		33% (1/3)	33% (1/3)	
Mean follow-up (years)		11 ± 3 (24)	10 ± 3 (20)	
Implant at follow-up	Standard implant	25% (6/24)	5% (1/20)	
	condylar constrained prosthesis	54% (13/24)	60% (12/20)	
	Hinged prosthesis	21% (5/24)	35% (7/20)	
HSS score		81 ± 10 (24)	63 ± 13 (4)	
	HSS objective	41 ± 5 (24)	40 ± 4 (4)	
	HSS symptoms	40 ± 8 (24)	38 ± 12 (20)	0.748
KSS objective score		80 ± 13.38 (24)	62 ± 22 (4)	
	KSS objective	64 ± 12 (24)	51 ± 24 (4)	
	KSS symptoms	15 ± 5 (24)	17 ± 7 (20)	0.338
KSS expectation and satisfaction score		34 ± 9 (24)	31 ± 14 (20)	0.346
	KSS satisfaction	24 ± 8 (24)	23 ± 10 (20)	0.884
	KSS expectation	10 ± 3 (24)	7 ± 4 (20)	0.062
KSS functional Activity score		52 ± 19 (24)	47 ± 22 (20)	0.524
	KSS walking and standing	19 ± 10 (24)	18 ± 10 (20)	0.468
	KSS standard activities	18 ± 6 (24)	17 ± 8 (20)	0.603
	KSS advanced activities	7 ± 6 (24)	7 ± 6 (20)	0.930
	KSS discretionary activities	8 ± 5 (24)	6 ± 4 (20)	0.256
OKS (12 = best result, 60 = worst result)		28 ± 6 (24)	32 ± 11 (20)	0.137
EQ-5D VAS		56 ± 21 (24)	49 ± 29 (20)	0.474
EQ-5D index (Reference: German value set)		0.694 ± 0.112 (24)	0.595 ± 0.218 (20)	0.123

ASA = American society of anesthesiologists; BMI = Body mass index; TKA = Total knee arthroplasty; rTKA = Revision total knee arthroplasty; ROM = Range of motion; HSS = Hospital for special surgery score; KSS = Knee society score; OKS = Oxford knee score; EQ-5D = Measure of health-related quality of life

**Table 3:** Demographic data, radiological and functional outcome aseptic vs. septic revisions

		Aseptic	Septic	*p*
N		32	12	
Gender	Female	56% (18/32)	33% (4/12)	
	Male	44% (14/32)	67% (8/12)	
Age at index surgery (years)		57 ± 8 (32)	61 ± 9 (12)	
Age at rTKA (years)		63 ± 8 (32)	64 ± 8 (12)	
Age at follow-up (years)		73 ± 9 (32)	75 ± 11 (12)	
ASA classification	ASA 1	0%	0%	
	ASA 2	79% (25/32)	67% (8/12)	
	ASA 3	21% (7/32)	33% (4/12)	
	ASA 4	0%	0%	
BMI (kg/m^2^)		29 ± 4 (32)	29 ± 6 (12)	
Affected side	Left	59% (19/32)	67% (8/12)	
	Right	41% (13/32)	33% (4/12)	
Time to revision after index TKA (years)		6 ± 6 (32)	3 ± 2 (12)	
Causes of rTKA	Loosening	50% (16/32)	0%	
	Infection	0%	100% (12/12)	
	Persistent pain	28% (9/32)	0%	
	Instability	13% (4/32)	0%	
	Component malrotation	9% (3/32)	0%	
Approach	One-stage	72% (23/32)	8% (1/12)	
	Two-stage	28% (9/32)	92% (11/12)	
Mean follow-up (years)		10 ± 3 (32)	11 ± 4 (12)	
Implant at follow-up	Standard implant	19% (6/32)	8% (1/12)	
	Condylar constrained prosthesis	56% (18/32)	59% (7/12)	
	Hinged prosthesis	25% (8/32)	33% (4/12)	
HSS score		78 ± 12 (27)	84 (1)	
	HSS objective	41 ± 5 (27)	44 (1)	
	HSS symptoms	39 ± 10 (32)	38 ± 8 (12)	0.625
KSS objective score		77 ± 16 (27)	85 (1)	
	KSS objective	62 ± 15 (27)	71 (1)	
	KSS symptoms	16 ± 6 (32)	17 ± 6 (12)	0.624
KSS expectation and satisfaction score		34 ± 10 (32)	29 ± 14 (12)	0.241
	KSS satisfaction	25 ± 8 (32)	22 ± 11 (12)	0.413
	KSS expectation	9 ± 3 (32)	7 ± 3 (12)	0.108
KSS functional activity score		51 ± 22 (32)	45 ± 13 (12)	0.275
	KSS walking and standing	19 ± 10 (32)	17 ± 7 (12)	0.381
	KSS standard activities	18 ± 7 (32)	16 ± 7 (12)	0.437
	KSS advanced activities	7 ± 6 (32)	5 ± 4 (12)	0.500
	KSS discretionary activities	7 ± 5 (32)	7 ± 3 (12)	0.850
OKS (12 = best result, 60 = worst result)		29 ± 9 (32)	33 ± 7 (12)	0.183
EQ-5D VAS		55 ± 24 (32)	48 ± 29 (12)	0.515
EQ-5D index (Reference: German value set)		0.661 ± 0.182 (32)	0.618 ± 0.151 (12)	0.322

ASA = American society of anesthesiologists; BMI = Body mass index; TKA = Total knee arthroplasty; rTKA = Revision total knee arthroplasty; ROM = Range of motion; HSS = Hospital for special surgery score; KSS = Knee society score; OKS = Oxford knee score; EQ-5D = Measure of health-related quality of life

The comparison of aseptic and septic revisions did not reveal significant differences regarding outcome scores as well. [Table 3; HSS symptoms score (*p* = 0.625), KSS symptoms score (*p* = 0.624), KSS expectation and satisfactions score (*p* = 0.241), KSS functional score (*p* = 0.275), OKS score (*p* = 0.183) and EQ-5D VAS level (*p* = 0.515)].

## Discussion

This study reports the indications, used implants, and outcomes of first revision TKAs in 44 patients treated between January 2000 and December 2012 at a university hospital. The mean follow-up time was 11 years, and the mean time between first TKA and revision TKA was five years. This is slightly lower than a mean of seven years to the first revision reported by previous studies [26, 31]. We could confirm our hypothesis that there is no differences in long-term outcome of one- and two-stage revision strategies as well as between septic and aseptic revisions. However, the latter results should be interpreted with caution with respect to the limited number of patients in the analyzed subgroups.

In the here presented population, the leading cause for revision surgery was aseptic loosening (36%) and periprosthetic joint infection (27%). This is in line with the findings by other published reports where both aseptic loosening and infection were listed among the first indications for rTKA [26, 28, 29]. The here presented cases show a high prevalence of infections and two-stage revisions which shows that rather complex cases where treated in our university center, while standard cases where rather treated in rural hospitals.

Complex cases with patients with higher comorbidities are associated with a higher risk of complications, prolonged hospitalization, and higher mortality [3, 16]. Treatment often requires a two-staged procedure, and the support of an interdisciplinary team usually only available in big centers [2].

The outcome scores and clinical results of this study are within the known range. Successful treatment thresholds of the KSS range between 72 and 86 one year after primary TKA [14]. After revision TKA, the KSS has been reported to range from 70 to 77 points [9, 22]. Our value of 77 points confirms these findings. The HSS score represents the satisfaction after TKA and has been reported to range between 83–90 points following primary TKA [25, 34]. Lee et al. [19] reported an HSS score of 79 after revision TKA due to periprosthetic joint infection and 85 in the aseptic treatment group. Our data indicate a similar outcome with an HSS value of 78 matching this range. The OKS score reported outcome scores following revision TKA range between 23 and 32 points [1, 4, 10, 21]. The here reported population reached a score of 30, indicating comparable patient satisfaction as previously reported. The here reported ROM was 114° while ranging from 76 to 112° in the literature [24].

This cohort reached a mean of 0.649 for the EQ-5D index and 53 for the EQ-5D VAS. Concerning the EQ-5D index, Baker et al*.* reported a score of 0.541 following revision TKA in a cohort of 797 patients. Like our cohort, all patients suffered from moderate to severe systemic diseases (ASA II–III, Table 1). While the mentioned group reported a mean follow-up of 7 months, our data represents a follow-up of 11 years. The extended time frame might explain the slightly superior outcome after revision TKA in our cohort because function and satisfaction relevantly improve during the first 12 months after surgery. Concerning the EQ-5D VAS values, no representative data are available. Therefore, our values are compared to the biggest neighbor-country Germany, where the age group of 65–74 years reaches an EQ-5D VAS score of 69 [15]. Compared to this population, the here reported cohort reached a 22% lower VAS score. However, looking at the ASA score, our cohort might not be comparable with Germany's average patient health status in that age group. Nevertheless, a lower VAS score is plausible in a cohort of patients with rTKA.

With respect to the subgroup comparison, we did not observe any significant functional differences between a one-stage and a two-stage approach following rTKA. While some authors report similar findings [24], others indicate superior functional outcomes following rTKA by a one-stage approach [23]. Therefore, ongoing randomized clinical trials aim to identify advantages of one-stage vs. two-stage strategies [20]. A two-stage procedure is traditionally favored for management of infected TKA. To analyze whether infection influences the outcome of such two-stage approaches, we compared two-stage rTKA due to infection vs. loosening. In doing so, we did not observe any significant differences between the two subgroups. Similar results have been reported in the past [33], but there is also some evidence showing worse results of infected rTKA compared to not infected rTKA [32]. This study has several limitations. Firstly, it is a small series of included patients, which makes subgroup analysis difficult. Secondly, the here reported population falls in the ASA II and III groups and might not represent patients’ average health status requiring a revision TKA. This possible selection bias might even be even intensified by the fact that rather complex cases are admitted to our university center, while standard case are treated in peripheral hospitals. Therefore, outcomes of rTKA in standard cases without significant comorbidities are assumed to be better than reported here. This might be the reason for the discrepancy in the literature while comparing outcomes of primary and revision TKA [30]. Finally, further interesting subgroup analysis regarding stability, ROM, loosening and function of the different implants could not be analyzed due to the limited number in each subgroup. A subgroup of patients was not willing to visit our outpatient department for physical and radiological examination at the final follow-up. Therefore, of this cohort only PROMs and survival could be raised. The reason for this is a long follow-up in an elderly cohort, for whom it is often a burden to attend medical appointments. Data of physical and radiological examination would have added some value, but the study question that focused on PROM and survival could be answered with the raised data. Despite these limitations, we believe that this work adds value to the scientific knowledge about rTKA because of the long follow-up period and the high number of PROMS obtained.

This study can be used to educate patients who are about to undergo the first revision of TKA and to inform them about the possible outcome. Despite a generally good outcome, patients have to be informed about the possibility of revision and/or implant removal, as this is very burdensome for those affected. Furthermore, given a non-significant difference between the outcomes of one- and two-stage-exchanges, the indication for a two-stage-exchange must be strictly defined. This is in concordance with a systematic review comparing one- and two-stage-exchange rTKA due to infection [23].

## Conclusion

In our 11 years follow-up, we obtained 89% implant survivorship. Measurements regarding functional outcome and pain showed results in the medium range of the respective scores, while patient satisfaction lay in the upper third. No significant differences in outcome scores between one- and two-stage revisions and septic versus aseptic revisions were observed.
